# PD-1 Blockade Can Restore Functions of T-Cells in Epstein-Barr Virus-Positive Diffuse Large B-Cell Lymphoma *In Vitro*

**DOI:** 10.1371/journal.pone.0136476

**Published:** 2015-09-11

**Authors:** Lina Quan, Xue Chen, Aichun Liu, Yan Zhang, Xiuchen Guo, Shujie Yan, Yue Liu

**Affiliations:** 1 Department of Hematology, Harbin Medical University Cancer Hospital, Harbin, Heilongjiang Province, China; 2 Department of Radiotherapy, Harbin Medical University Cancer Hospital, Harbin, Heilongjiang Province, China; University of Nebraska - Lincoln, UNITED STATES

## Abstract

Epstein–Barr virus-positive diffuse large B-cell lymphoma (EBV+DLBCL) is an aggressive malignancy that is largely resistant to current therapeutic regimens, and is an attractive target for immune-based therapies. Anti-programmed death-1 (PD-1) antibodies showed encouraging anti-tumor effects in both preclinical models and advanced solid and hematological malignancies, but its efficacy against EBV+DLBCL is unknown. Herein, we performed experiments using co-culture system with T cells and lymphoma cell lines including EBV+DLBCL and EBV-DLBCL [including germinal center B-cell like (GCB)-DLBCL and non-GCB-DLBCL] in vitro. We show that lymphoma cells augmented the expression of PD-1 on T cells, decreased the proliferation of T cells, and altered the secretion of multiple cytokines. However, through PD-1 blockade, these functions could be largely restored. Notbaly, the effect of PD-1 blockade on antitumor immunity was more effective in EBV+DLBCL than that in EBV-DLBCL in vitro. These results suggest that T-cell exhaustion and immune escape in microenvironment is one of the mechanisms underlying DLBCL; and PD-1 blockade could present as a efficacious immunotherapeutic treatment for EBV+DLBCL.

## Introduction

The immune system plays an important role in the development of cancer [1,2] including hematologic malignancies [3]. Epstein–Barr virus-associated diffuse large B-cell lymphoma (EBV+DLBCL) is an aggressive malignancy that is largely resistant to current therapeutic regimens and is an attractive target for immune-based therapies [4]. However, the efficacy of immune-targeted therapies in virus-related lymphomas has not been rigorously tested. Especially, the applicability of programmed death-1 (PD-1) blockade in the treatment of EBV+DLBCL has not been investigated so far.

PD-1 is a member of the B7 receptor family, which plays an important role in the regulation of immune response [5]. The PD-1 receptor, in conjunction with ligands PD-LI and PD-L2, regulates the immune response primarily by downregulating the signals of the T-cell receptor [3]. In inflammatory conditions (e.g., chronic infections), the sustained expression of PD-1 results in T-cell exhaustion and immune escape [6,7]. Similarly, tumors have adopted this mechanism to escape the antitumor activity of tumor-infiltrating lymphocytes that are present in the microenvironment [8]. In the case of tumor, the chronic antigen exposure persistently elevated the level of PD-1 which results in the exhaustion of antigen-specific T cells. PD-1 is expressed by tumor-infiltrating lymphocytes in the microenvironment in several hematologic malignancies including follicular lymphoma (FL), DLBCL, and classical Hodgkin lymphoma(cHL) [9–11]. As a newly emerged mechanism of tumor evasion from the antitumor immune response, PD-1 blockade results in, as expected, the re-establishment of the immune antitumor response [12]. Treatment strategies that block the PD-1 pathway are currently under development and recent clinical trials have shown clinical responses in a variety of solid tumors and some hematologic malignancies. Correlative studies from recent clinical trials of the PD-1 pathway blockade in FL and DLBCL after autologous stem-cell transplantation have generated encouraging results [13,14], which support the inhibition of immune checkpoint as a therapeutic mechanism.

Compared to solid tumors, the spectrum of expression of PD-L1 in lymphomas is not so wide [15]. Among B-cell lymphomas, the expression of PD-L1 is essentially confined to a subset of the clinically important activated B-cell (ABC)/non-germinal-center B (non-GCB) subtype of DLBCL, EBV-positive and -negative post-transplantation lymphoproliferative disorders, and EBV-associated DLBCL [16]. Within these tumors, PD-L1 was generally overexpressed by malignant cells and tumor-infiltrating macrophages [17]. The status of receptors present on both tumor cells or stromal cell in the tumor microenvironment is worthy of more exploration in order to better select disease-specific application of the anti-PD-1 agents. Choosing the appropriate tumor types is crucial for proving the applicability of PD-1/PD-L1 blockade in the treatment of hematologic malignancies.

In this study, we for the first time show that the number of effector/memory T cells and PD-1-positive cells infiltrating the DLBCL (EBV+ and EBV-) is higher than their counterparts in the peripheral blood, indicating the immune inhibition or immune escape in tumor microenvironment of DLBCL including both EBV-positive and -negative DLBCL. After screening of a panel of cell lines and human primary tumor samples, we detect the expression of PD-L1 on EBV-positive cell lines, non-GCB (ABC)-DLBCL cell lines, primary EBV-positive DLBCL, and non-GCB (ABC)-DLBCL tissue specimens using flow cytometry. Using allogenic co-culture system, we further show that lymphoma cells augment the expression of PD-1 on T cells, decrease the proliferation of T cells, decrease the secretion of interleukin-2 (IL-2), interferon-gamma (IFN-γ), tumor necrosis factor-alpha (TNF-α), and IL-10 in the supernatant of co-culture. Through PD-1 blockade, proliferation of T cells is increased, IL-2, IFN-γ, TNF-α, and IL-10 restored. We also find that the effects of PD-1 blockade on antitumor immunity are more effective in EBV+DLBCL than that in EBV-DLBCL, thus PD-1 blockade could restore immune escape resulting in more efficient T-cell exhaustion in EBV+DLBCL. These results suggest that PD-1 blockade presents a more efficacious immunotherapy to EBV+DLBCL.

## Materials and Methods

### Cell lines

EBV–transformed lymphoblastoid B-cell lines (LCL) was developed in our laboratory as described previously [18]. Three lines of LCLs were established from patients with EBV+DLBCL (LCL-1), EBV-DLBCL (LCL-2), and a healthy subject (LCL-3), respectively. In brief, peripheral blood samples were collected and used to generate LCL. Concentrated supernatant from B95-8 cultures, a EBV-transformed marmoset B-cell line, was added to parallel samples in the presence of cyclosporin A in RPMI 1640/20% FCS.

SU-DHL-4, SU-DHL-6, HBL-1, and OCI-Ly-10 DLBCL cell lines were gifts from Dr. Ronald Levy (Stanford University, Stanford, CA, USA). Raji and Daudi human Burkitt lymphoma (BL), Karpas 299 anaplastic large cell lymphoma (ALCL) and Jurkat T-cell lymphoblastic leukemia cell lines were obtained from the American Type Culture Collection (ATCC).

SU-DHL-4, HBL-1, LCL, Raji, Daudi, Karpas 299, and Jurkat cells were cultured in RPMI 1640 medium (Invitrogen, Carlsbad, CA, USA) plus 10% heat-inactivated fetal calf serum (FCS; Omega Scientific, Tarzana, CA, USA), 100 U/mL penicillin/streptomycin, and 2 mmol/L L-glutamine, at 37°C in 5% CO_2_. SU-DHL-6 cells were cultured in RPMI 1640 medium plus 20% FCS. OCI-Ly-10 cells were cultured in Iscove’s Modified Dulbecco’s Media(IMDM) complete medium plus 20% fresh human plasma (heparinized) instead of FCS.

### Preparation of clinical samples

Matched human primary lymphoma specimens and peripheral blood (PB) (n = 27) including 8 GCB-DLBCL, 12 ABC-DLBCL, and 7 EBV+DLBCL (Table 1) were obtained from previously untreated patients with written informed consent according to the Declaration of Helsinki and the study protocol (KY2015-02) was approved by the Clinical Research Ethics Committee of The Cancer Hospital of Harbin Medical University (Harbin, China). PBMCs from healthy donors were used as controls (n = 15).

**Table 1:** Patients’ characteristics of the study population according to EBV-encoded RNA (EBER) status.

Patients’ characteristics	All the cases Number of cases(%)	EBV positive DLBCL Number of cases(%)	EBV negative DLBCL Number of cases(%)	*P*
**Number of patients**	27(100)	7(26)	20(74)	
**Gender**				
Male	16(59)	5(71)	11(55)	
Female	11(41)	2(29)	9(45)	
***IPI factors***				
**Age (years)**				
≤60	15(56)	2(29)	13(46)	
>60	12(44)	5(71)	7(54)	<0.05
**Performance status**				
ECOG PS (0–1)	13(48)	2(29)	11(55)	
ECOG PS (2–4)	14(52)	5(71)	9(45)	<0.05
**LDH level**				
low	13(48)	2(29)	11(55)	
high	14(52)	5(71)	9(45)	<0.05
**Ann Arbor Stage**				
Limited, I-II	16(59)	3(43)	14(70)	
Advanced, III-IV	11(41)	4(57)	6(30)	<0.05
**Extranodal site involvement**				
0 or 1 site	17(63)	4(57)	13(65)	
2 and more sites	10(37)	3(43)	7(35)	
***IPI score (number of IPI factors)***				
Low risk(0,1) and low-intermediate risk(2)	15(56)	2(29)	13(65)	
High-intermediate risk(3) and high risk(4,5)	12(44)	5(71)	7(35)	<0.05
**B symptom**				
Positive	9(33)	4(57)	5(25)	
Negative	18(67)	3(43)	15(75)	<0.05
**Bone marrow involvement**				
Positive	9(33)	3(43)	6(30)	
Negative	18(67)	4(57)	14(70)	
			Subtype: Number of cases /total number of cases (%)	
			GCB-DLBCL: 8/20(40)	
			ABC-DLBCL: 12/20(60)	

Abbreviations: ABC: activated B-cell-like type DLBCL; GCB: germinal center B-cell-type DLBCL; IPI: International Prognosis Index.

Diagnoses were made from biopsies taken from the primary tumors and cases were classified as DLBCL according to the WHO scheme for B-cell non-Hodgkin’s lymphoma (NHL) [19]. Cases were then subcategorized as GCB or non-GCB according to the algorithm presented previously [20]. To determine the EBV status, EBV-encoded small nuclear RNA(EBER)(Leica Microsystems, UK) in situ hybridization (ISH) was performed on all the cases. To further determine the EBV latency types, LMP1(CS1-4, Dako, Germany), LMP2A (15F9, AbCam, UK), EBNA2(PE2, AbCam, UK) and EBNA3A(ab16126, Abcam, UK) were detected on EBER positive cases by immunohistochemistry (IHC), respectively.

Primary lymphoma biopsy specimens from patients with DLBCL were gently minced over a wire mesh screen to obtain a signle cell suspension. Peripheral blood mononuclear cell (PBMC) was enriched from peripheral blood using Ficoll–Hypaque sedimentation (Sigma, St Louis, MO, USA). Cell suspension of lymph node (LN) and PBMC was either analyzed directly or cryopreserved in liquid nitrogen. For analysis, cryopreserved specimens were thawed quickly in a 37°C water bath and washed twice with warm RPMI complete medium before use.

### Flow cytometry analysis

Monoclonal antibodies (mAbs) used to measure the expression of cell surface markers using flow cytometry included CD3, CD4, CD8, CD10, CD19, CD45RA, CD45RO, CD62L, PD-1, PD-L1, CTLA-4, and appropriate isotype controls. PD-1 antibody was purchased from Miltenyi Biotec (Bergisch Gladbach, Germany) and others were all from Becton Dickinson(BD) Biosciences (Mansfield, MS,USA) or BD Pharmagen (San Diego, CA,USA). Stained cells were analyzed using a BD FACS CantoII flow cytometer (BD Biosciences) with Diva software (BD Biosciences).

### Allogeneic T-cell proliferation assay

T cells were enriched from whole blood obtained from healthy donors with informed consent, using positive selection with the CD3 microbeads (Miltenyi Biotec) according to the manufacturer’s protocol. The cells were routinely analyzed using flow cytometry, and the purity of T populations was >96%. A portion of the enriched cells were labeled with 1 μM carboxyfluorescein succinimidyl ester (CFSE) (Sigma) for 12 min at 37°C then washed twice with phosphate-buffered saline. Another portion of unlabeled enriched cells was used for analyzing cytokine secretion. CFSE-labeled T cells (2x10^5^) were cultured in RPMI complete medium at a 10:1 ratio with irradiated (3000 R) LCL cells or irradiated (3000 R) OCI-Ly-10 cells or irradiated (3000 R) SU-DHL-4 cells. Cells were fed every 2 days with a fresh medium containing 10 IU/mL IL-2 (Peprotech, Rocky Hill, NJ, USA). After 1 week, T cells were harvested, counted, and replated in quadruplicate with irradiated (3000 R) LCL, OCI-Ly-10, or SU-DHL-4 cells, respectively, at effector:target (E:T) ratio of 2:1 with 2x10^4^ tumor cells per well in 96-well U-bottom plates with or without 10 μg/mL anti-PD-1 (J116) or immunoglobulin G1 (IgG1) isotype control mAbs (eBioscience, San Diego, CA, USA). On day 4, 10 μg/mL anti-PD-1 or IgG1 isotype control mAbs was replenished, cells were harvested after 16 h, stained with CD3 (BD Biosciences), and proliferation was analyzed using flow cytometer and software described earlier.

### Cytokine analyses using cytometric bead array

For allogeneic experiments, enriched T cells unlabeled CFSE (2x10^5^) were cultured in RPMI complete medium at a 10:1 ratio with irradiated (3000 R) LCL cells, or irradiated (3000 R) OCI-Ly-10 or irradiated (3000 R) SU-DHL-4 cells. Cells were fed every 2 days with a fresh medium containing 10 IU/mL IL-2 (Peprotech). After 1 week, T cells were harvested, counted, and replated in quadruplicate with irradiated (3000 R) LCL cells, OCI-Ly-10 cells or SU-DHL-4 cells, respectively, at E:T ratio of 2:1 with 2x10^4^ tumor cells per well in 96-well U-bottom plates added phytohemagglutinin (PHA) at 2 μg/mL, with or without 10 μg/mL anti-PD-1 (J116) or IgG1 isotype control mAbs (eBioscience). Supernatants from each group were collected after 5 days of incubation and analyzed for IL-2, IL-4, IL-6, IFN-γ, IL-10, and TNF-α using flow cytometric bead array (CBA) Th1/Th2 cytokine kit (BD Pharmagen), and using a BD FACS CantoII flow cytometer (BD Biosciences) with Diva software (BD Biosciences). Data were analyzed using FCAP Array V3 software.

### Statistical analysis

Data are presented as mean±standard deviation. SPSS 17.0 software and independent-sample t test or one-way analysis of variance were used for statistical analysis. *P*-value<0.05 was considered statistically significant(*P<0.05, **P<0.01 and ***P<0.001).

## Results

### Characteristics of the study population

Characteristics of the study population (n = 27) were summarized in Table 1. Based on the detectable level of EBER, 20 were EBER negative cases in which 8(40%) cases were classified into GCB subtype and 12 (60%) non-GCB(ABC) subtype according to IHC classification. Compared to EBV-negative DLBCL, patients with EBV-positive DLBCL had higher age (median, 67 versus 54 years) and a closer association with aggressive clinical features or parameters: 5(71%) patients older than 60, 5(71%) with performance status (PS) >1, 5(71%) with high level serum lactate dehydrogenase (LDH), 4(57%) with advanced stage (III-IV) at diagnosis, and 4(57%) with B symptoms. As a result, the International Prognostic Index (IPI) score for patients with EBV+DLBCL was significantly higher than that for patients with EBV-DLBCL, with 5(71%) of the EBV-positive group categorized in the IPI high intermediate-risk and high group. There was no statistical difference between two groups in the incidence of having more than one extranodal site and bone marrow involvement. These results were consistent with previous study [21,22], although the number of cases in this study was fewer.

Regarding the EBV latency status, among 7 EBER positive DLBCL, 5 cases were latency type III and 2 cases were latency type II/III. The expression of LMP1, LMP2A, EBNA2 and EBNA3A in EBV positive cases were summarized in S1 Table. It is well known that LCL is typical latency III, therefore we used LCL to simulate tumor cells of EBV+DLBCL in our following experiments.

### PD-L1 is expressed by ALCL, EBV-positive cell lines, ABC (non-GCB) DLBCL cell lines but not by GCB-DLBCL cell lines

PD-1 is a receptor and its proper function involves ligands (i.e., PD-L1 and PD-L2). In order to find a suitable experimental model as well as experimental strategy to test PD-1 blockade as an immunotherapeutic method, we first screened a panel of human lymphoma cell lines for the expression of PD-L1 using flow cytometry (Table 2). Representative histograms are shown in S1 Fig. Among DLBCL cell lines, ABC-DLBCL cell lines HBL-1 and OCI-Ly-10 were positive for PD-L1, whereas GCB-DLBCL cell lines (SU-DHL-4 and SU-DHL-6) were negative for PD-L1. Three types of EBV+LCL developed from patient with EBV+DLBCL, EBV-DLBCL, and healthy subject, respectively, were all positive for PD-L1, and there was no difference among different origins for the expression of PD-L1. ALCL cell line Karpas 299 and T- acute lymphoblastic leukaemia (T-ALL) cell line Jurkat were positive for PD-L1. Burkitt cell lines Daudi and Raji were negative for PD-L1.

**Table 2:** Expression of PD-L1 among a panel of lymphoma cell lines.

Cell Line	Lymphoma subtype	PD-L1
LCL-1	EBV+	+
LCL-2	EBV+	+
LCL-3	EBV+	+
SU-DHL-4	DLBCL(GCB)	-
SU-DHL-6	DLBCL(GCB)	-
OCI-Ly-10	DLBCL(ABC)	+
HBL-1	DLBCL(ABC)	+
Daudi	Burkitt	-
Raji	Burkitt	-
Karpas 299	ALCL	+
Jurkat	T-ALL	+

Note:Expression of PD-L1 was measured by flow cytometry.”+” indicates > 2 log MFI above isotype control; “+”indicates < 2 log MFI above control.

### PD-L1 is expressed by a subset of human primary DLBCLs

We next tested the human DLBCL tissue specimens for the expression of PD-L1. DLBCL was classified into GCB or non-GCB subtype based on immunohistochemical analysis of markers CD10, Bcl-6, and MUM-1, which correlate with cell origin subtypes as determined using gene expression profiling. Single-cell suspensions of 27 DLBCL, including 8 GCB-DLBCL, 12 ABC-DLBCL (non-GCB-DLBCL), and 7 EBV+DLBCL, were analyzed using flow cytometry. Expression of PD-L1 among these DLBCL was heterogeneous (Table 3) with 48% of DLBCL specimens showing expression of PD-L1. Eight of 12 ABC-DLBCL specimens expressed PD-L1 on malignant B cells. None of the 8 GCB-DLBCL specimens were positive for PD-L1. Among the seven EBV+DLBCL specimens, five expressed PD-L1 (shown in S2 Fig). This pattern of expression parallels the results in cell lines, where the expression of PD-L1 was found in ABC-DLBCL and EBV+LCL, but none of GCB-DLBCL cell lines.

**Table 3:** Expression of PD-L1 in primary DLBCL tissue specimens.

PD-L1 detection	DLBCL subtype	Number of cases	Number of PD-L1+	%PD-L1+
**Flow cytometry**	GCB-DLBCL	8	0	0
	ABC(non-GCB)-DLBCL	12	8	66.7
	EBV+DLBCL	7	5	71.4

Note: PD-L1 expression in DLBCL occurs almost exclusively in tumors of non-germinal center origin and EBV positive DLBCL.

### T cells in DLBCL microenvironment display effector memory phenotypic traits with increased expression of PD-1

**Fig 1:**
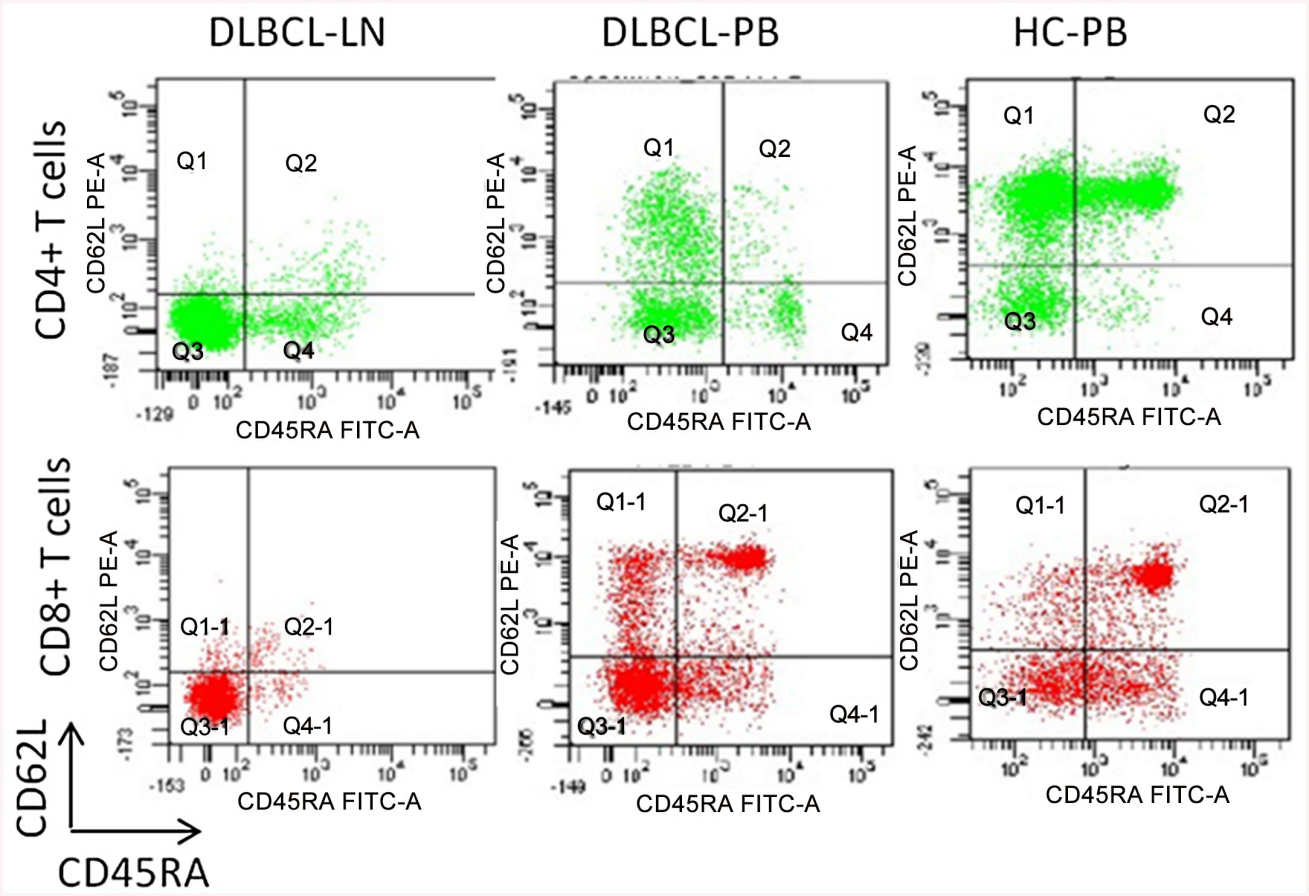
The comparison of effector memory T cells (CD62L-CD45RA-) in lymph node (LN) and peripheral blood (PB) of DLBCL and peripheral blood (PB) of healthy controls (HC). Dot plot shows CD45RA-CD62L- effector memory CD3+CD4+ and CD3+CD8+ T cells in LN and PB of a DLBCL patient and PB of a healthy person.

**Fig 2:**
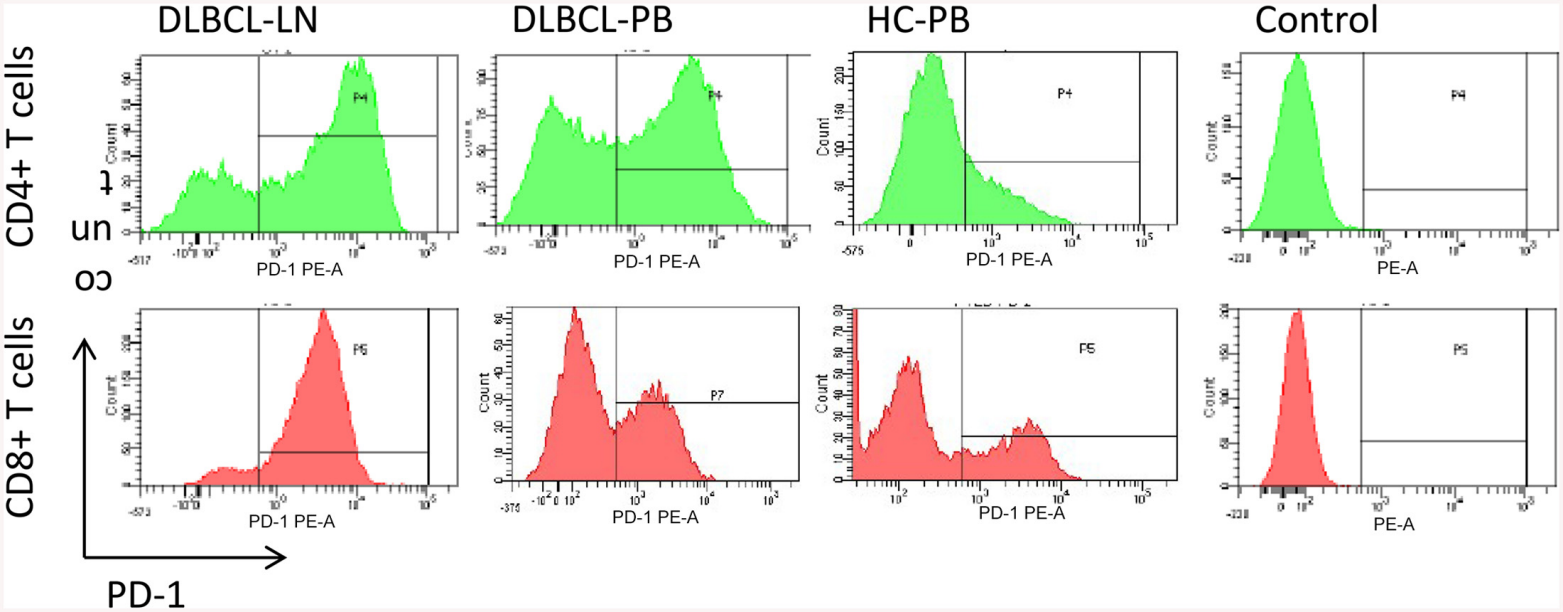
The comparison of PD-1 expression in lymph node (LN) and peripheral blood(PB) of patients of DLBCL and peripheral blood(PB) of healthy controls (HC). Shown are the expression of PD-1 on CD4+ and CD8+ T cells in LN and PB of a DLBCL patient and a healthy person.

To investigate the state of differentiation of T cells and expression of markers characteristic of chronic antigen-specific stimulation in the microenvironment of DLBCL, we characterized the phenotype of T cells from 27 DLBCL including 8 GCB-DLBCL, 12 ABC-DLBCL (non-GCB-DLBCL), and 7 EBV+DLBCL, and 15 healthy control subjects. Cell surface expression of CD3, CD4, and CD8, differentiation markers CD62L and CD45RA, and inhibitory receptors PD-1 and CTLA-4 were measured. A comparison of the phenotypic traits of T cells in LN and PB is summarized in Tables 4 and 5. The original results derived from all the patients were shown in S2, S3 and S4 Tables. T cells in LN showed a higher frequency of effector memory(Tem) cells (CD62L-CD45RA-) compared with counterparts in PB obtained from 27 patients tested and 15 healthy control subjects (Fig 1). In addition, T cells in LN exhibited enhanced expression of PD-1 compared with counterparts in PB obtained from patients and healthy control subjects (Fig 2). The ratio of Tem cells and the expression of PD-1 showed no significant difference in the subtypes of DLBCL.

**Table 4:** The ratio of CD4+ and CD8+ effector T cells in DLBCL primary tissue and peripheral blood.

	GCB-DLBCL (n = 8)		ABC-DLBCL (n = 12)		EBV+DLBCL (n = 7)		Healthy control (n = 15)
	LN	PB	LN	PB	LN	PB	PB
CD4 Tem/CD4+T(%)	61.20±6.13***	32.30±5.15	63.91±13.20***	35.71±8.19	71.73±15.12***	43.00±7.41	23.31±4.50
CD8 Tem/CD8+T(%)	65.20±8.14***	36.50±9.15	68.22±12.00***	39.69±6.79	79.17±11.89***	44.30±7.83	28.40±6.78

**Note:** LN: lymph node, PB: peripheral blood, DLBCL: diffuse large B-cell lymphoma, Tem: effector T cells

***P<0.001

**Table 5:** The ratio of PD-1 expression (%) on CD4+and CD8+ T cells in DLBCL primary tissue and peripheral blood.

	GCB-DLBCL (n = 8)		ABC-DLBCL (n = 12)		EBV+DLBCL (n = 7)		Healthy control (n = 15)
	LN	PB	LN	PB	LN	PB	PB
PD-1/CD4+T cells (%)	54.79±8.85***	37.60±8.35	58.91±13.20***	43.71±8.19	64.60±10.81***	39.4±7.73	13.31±4.50
PD-1/CD8+T cells (%)	62.25±11.55***	40.48±6.95	68.22±12.00***	49.69±6.78	74.63±11.56***	46.08±7.78	19.40±6.78

**Note:**LN: lymph node, PB: peripheral blood, DLBCL: diffuse large B-cell lymphoma, Tem: effector T cells

***P<0.001

**Fig 3:**
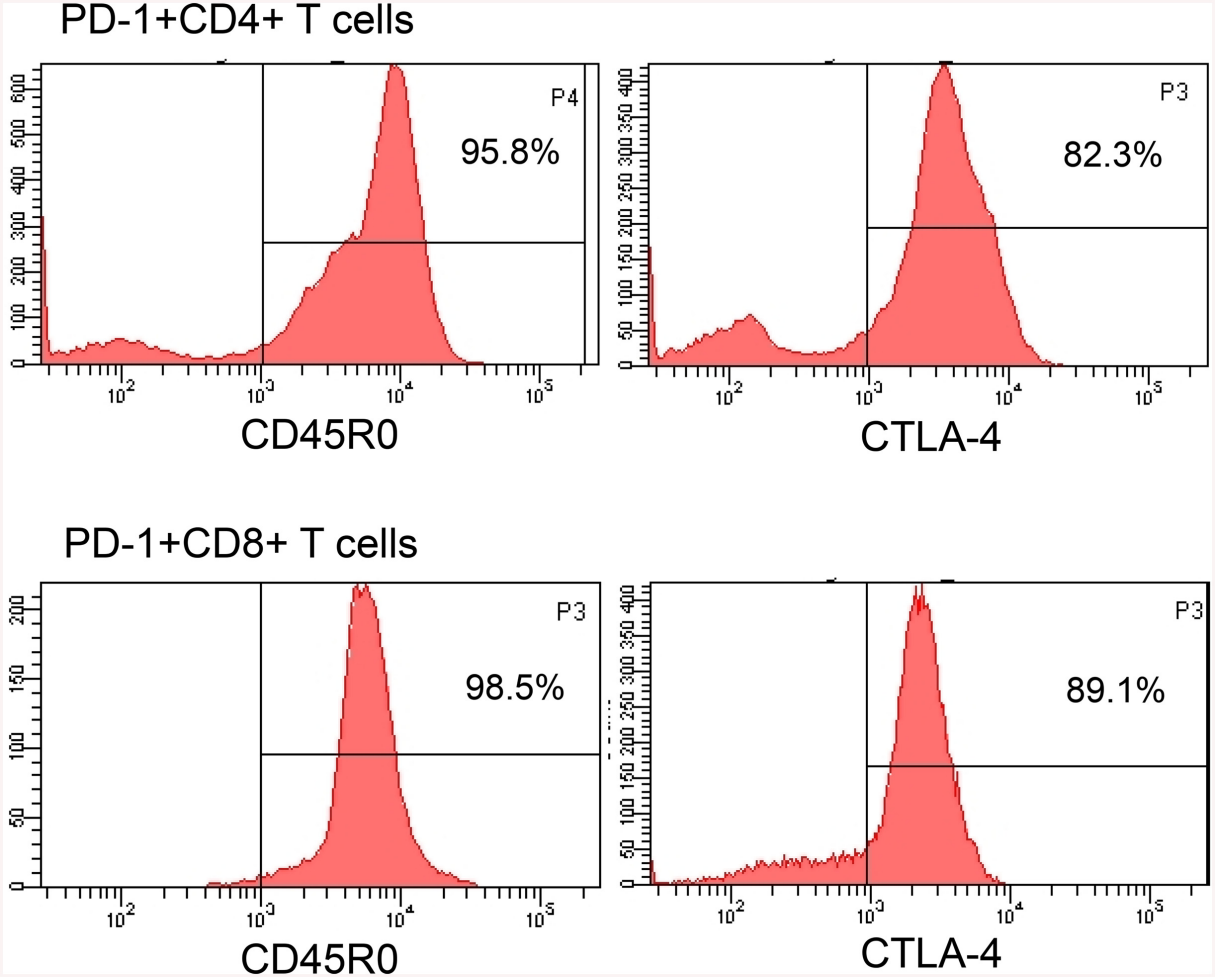
PD-1 positive T cells are exhausted T cells. PD-1 positive T cells express memory marker CD45RO and exhaustion markers CTLA-4. Histograms showing CD45RO and CTLA-4 on intratumoral preexisting CD4+ PD-1+ or CD8+ PD-1+ T cells from DLBCL.

To phenotypically characterize PD-1+ T cells, the surface markers on PD-1+ T cells were measured. Intratumoral PD-1+ T cells expressed high levels of CD45RO, indicating that the cells were memory T (Tm) cells. Most CD8+PD-1+ and some CD4+PD-1+ T cells expressed CTLA-4 (Fig 3), indicating that PD-1+ T cells are exhausted T cells.

### Tumor cells augment the expression of PD-1 in activated T cells

**Fig 4:**
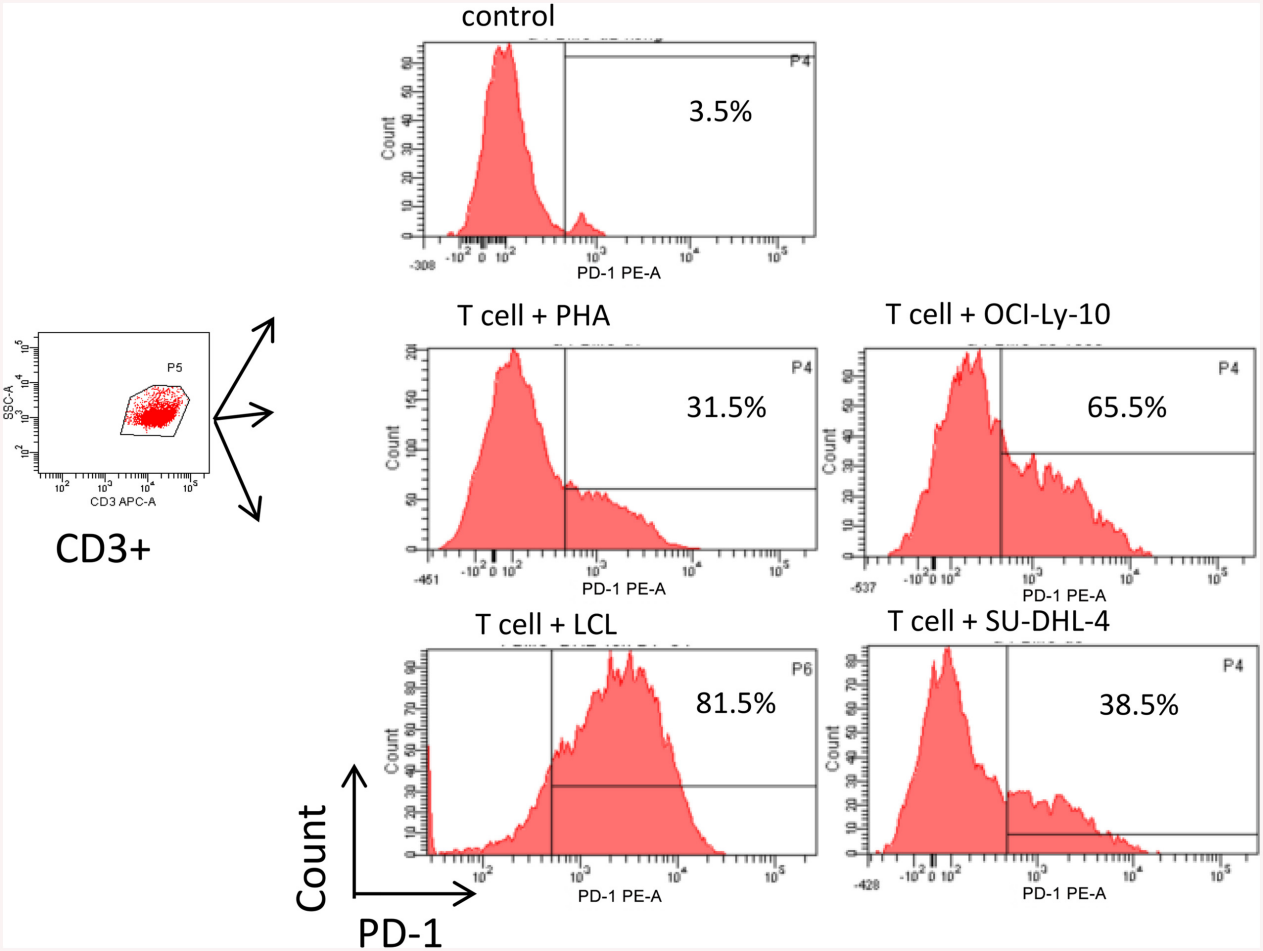
Tumor cells augment the PD-1 expression in activated T cells. Shown are CD3-positive-gated and the histograms of PD-1 expression on resting T cells, T cells activated by PHA after 24h, T cells co-cultured with OCI-L-y-10, LCL, and SU-DHL-4 overnight from a representative experiment (n = 4).

Based on the above results that tumor microenvironment T cells have elevated expression of PD-1, we reasoned that tumor cells might be the stimuli to upregulate PD-1 in T cells. We have previously established a B-LCL cell line from a patient with EBV+DLBCL, which could be used as a stimulating EBV+DLBCL tumor cells. To explore the expression of PD-1 in T cells after activation experimentally, we cultured T cells with or without PHA for 24 h and then analyzed the expression of PD-1 using FACS. The expression of PD-1 was at a low level (about 3.5% expression) on resting T cells. In contrast, a significant increases expression (about 35.2%) of this molecule was observed after T cells were activated (n = 4) (Fig 4). To further analyze the effect of tumor cells on the expression of PD-1 by activated T cells, activated T cells were co-cultured with B-LCL, OCI-Ly-10, and SU-DHL-4 overnight, respectively. Higher expression levels of PD-1 were found in the co-cultures as compared with activated T cells alone (n = 4). Moreover, activated T cells co-cultured with B-LCL and OCI-Ly-10 exerted higher expression of PD-1 than those co-cultured with SU-DHL-4 (Fig 4, and Table 6). As shown above (Table 1), PD-L1 was expressed on B-LCL and OCI-Ly-10 but not SU-DHL-4, implying that tumor cells may interact with T cells via PD-1/PD-L1 signaling.

**Table 6:** The ratio of PD-1 expression (%) on resting T cells, T cells activated by PHA after 24h, T cells co-cultured by normal B cells, LCL, and SU-DHL-4 overnight. Activated T cells co-cultured with B-LCL and OCI-Ly-10 exerted higher expression of PD-1 than those co-cultured with SU-DHL-4.

	control	PHA	OCI-Ly-10	LCL	SU-DLH-4
PD-1 expression (%)	5.30±2.06	35.20±3.52	68.63±3.56***	75.43±5.27***	47.13±8.19
n = 4			*P<*0.001	*P*<0.001	

Note:

***P<0.001

### PD-1 blockade restores T-cell proliferation more effectively in PD-L1-expressed DLBCL subtype

**Fig 5:**
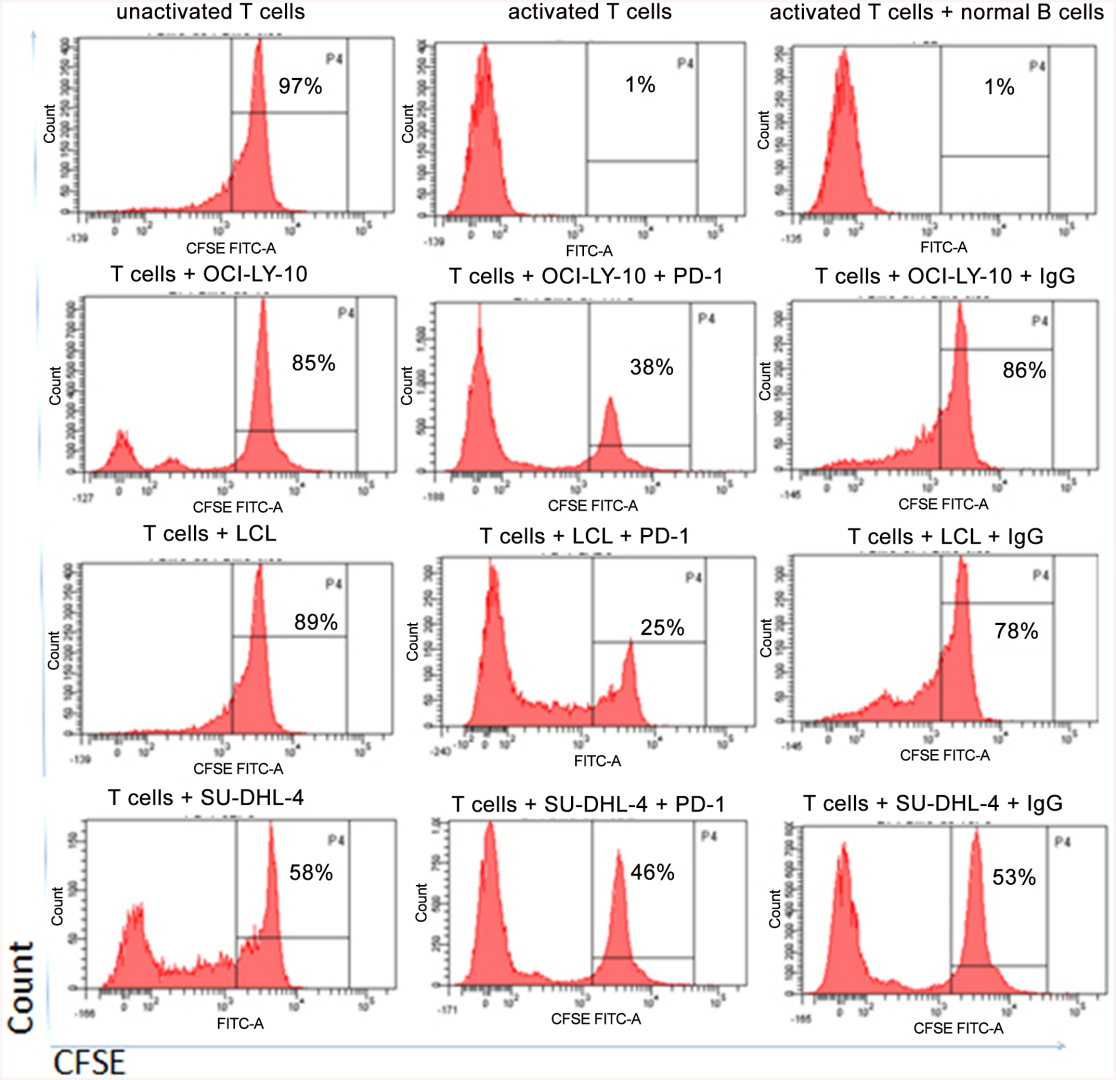
PD-1 blockade restores the proliferation of exhausted T cell resulted by lymphoma cells. T cells were labeled with CSFE and stimulated with OCI-Ly-10 cells, LCL cells and SU-DHL-4 cells, respectively. Percentages indicate the fraction of cells in the non-dividing peak. Shown are CD3-positive-gated histograms from a representative experiment (n = 4). Tumor cells inhibite T cell proliferation as observed by CSFE staining, and through PD-1 blockade T cell proliferation restored.

Considering that the expression of PD-1 was elevated on the T cells of the DLBCL tissues and the expression of PD-L1 was elevated in certian subtypes of DLBCL specimens (ABC-DLBCL and EBV+DLBCL) and cell lines, we speculated that disruption of PD-1/PD-L1 interaction might restore normal function of T cells. To stimulate the tumor microenvironment in vivo of DLBCL, allogeneic T cells from healthy donors were stimulated by irradiated B-LCL, OCI-Ly-10, or SU-DHL-4 in the ratio of 10:1 for a week, and co-culture experiments of activated T cells with B-LCL, OCI-Ly-10, and SU-DHL-4 were established, respectively. To investigate how this different expression of PD-L1 on B-LCL, OCI-Ly-10, and SU-DHL-4 would contribute to the effects of PD-1 blockade, proliferation experiment was performed using CFSE-labeled T cells as described [23]. As shown in Fig 5 and Table 7, a significant inhibitory effect on T cells proliferation was identified and this inhibitory effect was significantly restored by the PD-1-blocking antibody, consistent with earlier reports [24]. However, we found that, surprisingly, PD-1 blockade restored the proliferation (% CFSE dim cells) of T cells more markedly in co-culture with B-LCL and OCI-Ly-10 than with SU-DHL-4 (Table 7). Collectively, PD-1 blockade re-established the proliferation activity of T cells more efficiently in EBV+DLBCL and ABC-DLBCL than in GCB-DLBCL.

**Table 7:** PD-1 blockade restored the proliferation of T cells (%CFSE dim cells).

CFSE dim (%)	T+LCL	T+OCI-Ly-10	T+SU-DHL-4
With PD-1 blockade	72.33±3.71***	62.71±4.75***	51.10±3.94
With IgG blockade	24.80±3.80	14.50±3.56	47.83±3.53
n = 4	*P<*0.001	*P<*0.001	*P>*0.5

Note:

***P<0.001

### PD-1 blockade has distinct effects on the production of cytokines in different subtypes of DLBCL

**Fig 6:**
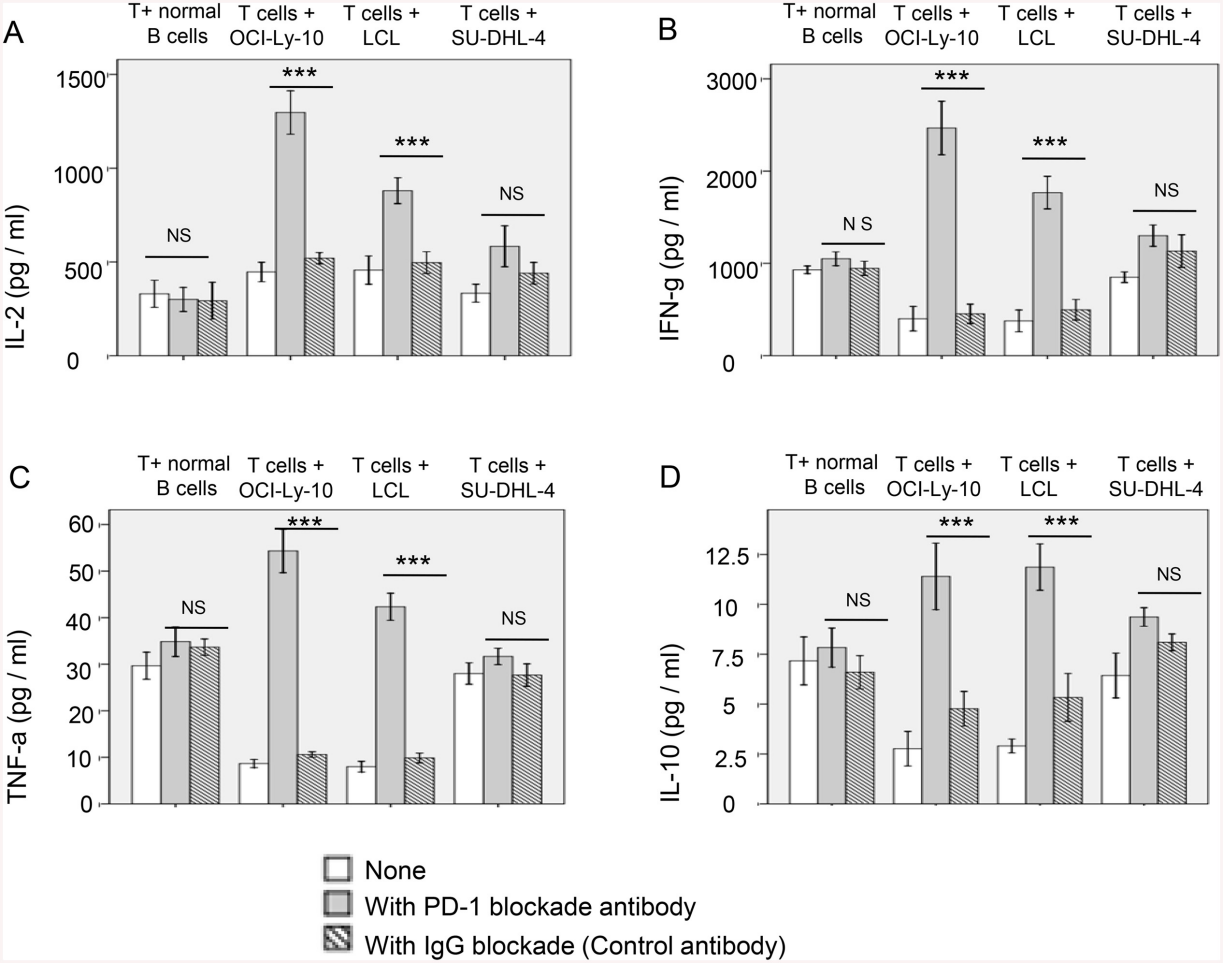
PD-1 blockade enhances the activation of T cells cocultured with allogeneic lymphoma cells (OCI-Ly-10, LCL and SU-DHL-4). PD-1 blockade affects T cells incubated with PD-L1 positive lymphoma cells (OCI-Ly-10 and LCL). However, incubation of healthy donor T cells with a B-cell lymphoma line that does not express PD-L1 (SU-DHL-4) does not significantly alter cytokine secretion with the blockade of PD-1. Data are represented as mean±SD of quadruplicate experiments. *P* values shown are for anti-PD-1 antibody versus isotype control antibody. (A) IL-2, (B) IFN-g, (C) TNF-a, (D) IL-10. ***P<0.001.

Based on our above observation that PD-1 was upregulated on activated T cells after being exposed to tumor cells expressing ligand PD-L1, we hypothesized that the interactions of tumor cells and activated T cells could influence the production of cytokines, such as IL-2, IFN-γ, IL-10, and TNF-α. Thus, the cytokines in the tumor cell-T cell co-culture systems were determined in the presence or absence of PD-1 mAb to investigate whether blockade of PD-1 signaling influenced the production of those cytokines. Supernatants were collected from co-culture systems for the analysis of cytokines using the CBA. Results showed that the production of cytokines in the co-culture systems was significantly altered after PD-1 blockade compared with those in the co-cultures without blockade (n = 4) (Fig 6 and S3 Fig). Interestingly, blockade of PD-1 pathway in PD-L1+ cell (e.g., B-LCL, OCI-Ly-10)-T cell co-culture system produced more IL-2, IFN-γ, IL-10, and TNF-α, than those in PD-L1- SU-DHL-4-T cell co-culture system, indicating a more dramastic effect of PD-1 blockade in EBV+DLBCL.

## Discussion

EBV-associated DLBCL is an aggressive malignancy that is largely resistant to current chemotherapeutic regimens. Given that its association with oncogenic virus, it is an attractive target for immune-based therapies. However, the efficacy of immune-targeted therapies in EBV-associated DLBCL has not been well tested. In this study, we examined the effect of PD-1 blockade on antitumor immunity in lymphoma cells, and found that PD-1 blockade exerted high efficacy in EBV+DLBCL.

It’s well established that PD-1 is a hallmark of chronically stimulated T cells during chronic infection or in the tumor microenvironment [25,26]. High PD1 expression is associated with T-cell exhaustion; blockade of the PD1/PD-ligand pathway with antibodies against PD1, PD-L1, or both, augmented or restored the function of viral-infection-specific and tumour-specific CD4+ T cells and CD8+ T cells in mouse and human studies [24]. In patients with lymphoma, therapeutic agents targeting immune checkpoints are expected to enhance endogenous antitumor immune responses and therefore benefit patients with pre-existing antitumor immunity [27]. Consistently, the data from the present study demonstrated that the effector/memory T cells were indeed expanded in DLBCL, indicative of existence of antitumor immunity in the microenvironment of DLBCL. In this study, we used FACS with combination of different markers (e.g, CD3, CD4, CD8, CD45RO, CTLA-4) to identify functional subtypes of T cells. Based on the augmented expression of PD-1 on CD4+ and CD8+ T cells, we found that these T cell populations were not only activated T cells but also exhausted T cells and memory T cells, as evidenced by the cell surface expressed of CD45RO and CTLA-4 (Fig 5). These results suggest that exhausted T cells exist in DLBCL regardless of EBV status, and highlight the PD-1 pathway as a potential mechanism underlying tumor evasion from the antitumor immune response in DLBCL.

The PD-1/PD-L1 pathway has been demonstrated in a broad spectrum of solid malignancies and hematologic malignancies including lymphomas and leukemia. PD-L1 overexpression in tumors is driven in part by chronic exposure to the pro-inflammatory cytokine IFN-γ and other inflammatory mediators [15,28]. Expression of PD-L1 on tumor cells has been reported to be associated with poor prognosis in many tumor types, demonstrating that immune tolerance mediated by the PD-1/PD-L1 pathway has clinical significance [29–31]. Some virus-associated cancers(including EBV-associated lymphoma [32]) were reported to induce PD-L1 expression [33–36], and it appears that infection-associated cancers could create an “immune-privileged” milieu by up-regulating PD-L1. In lymphoma, PD-L1 expression is often driven by intrinsic genetic aberrations and disregulated signaling pathways within malignant cells. Furthermore, in Hodgkin lymphoma, chromosomal abnormalities of 9p24.1,a genomic region that includes CD274, PDCD1LG2 (encoding PD-L), and Janus kinase 2(JAK2), have been correlated with increased PD-L1 expression [37]. A subset of cHL is EBV positive, and aberrant signaling through EBV-encoded gene products provides alternative mechanisms to upregulate PD-L1 [32]. In a genetically annotated series of primary cHL, 9p24.1 amplification and EBV-infection were mutually exclusive [38]. Similarly, primary mediastinal large B-cell lymphoma(PMLBCLs) also show gains in 9p24.1 [37]. In EBV-transformed human B-cell lines (lymphoblastoid cell lines, LCLs), LMP-1 promotes both AP1-signaling and JAK-STAT signaling to activate the enhancer and promoter elements of CD274, respectively [32]. Extended chromosome 9p24.1 amplification also includes a JAK2 locus that increases protein expression and activity [39,40]. In EBV-positive DLBCL, the virus maintains type II or type III latency program [41], suggesting that LMP1-mediated signaling is likely to directly contribute to tumorigenesis and to the immune evasion signature. Collectively, the levels of PD-L1 expression on tumor cells vary broadly, and the underlying mechanisms of PD-L1 up-regulation in EBV-associated lymphoma has not been fully elucidated, although there are some previous studies.

Considering that the expression of PD-L1 is currently a determinant of the efficacy of PD-1 mono-antibody treatment, it would be crucial to study whether the low expression or even absence of PD-L1 expression is associated with less effective therapy (see below). We investigated the expression of PD-L1 among a panel of lymphoma cell lines and primary lymphoma specimens. Interestingly, we show that the expression of PD-L1 is confined to a subset of the EBV+DLBCL and non-GCB(ABC) of DLBCL using FACS. Our results are consistent with previous studies using Immunohistochemistry (IHC)[16]. Based on this expression pattern, we speculate that PD-1 blockade will exert differential effects on DLBCL subtypes. As expected, PD-1 blockade exerted high efficacy in EBV+DLBCL compared to EBV-DLBCL. Mechanistically, we show that EBV+LCL can engage PD-1+ T cells through its ligand PD-L1 expression, and further inhibit T-cell proliferation and production of cytokines. Thus, PD-1 blockade could restore, at least partially, the antitumor immunity of T-cells. To the best of our knowledge, this is the first study to analyze the effects of the PD-1 blockade on EBV+DLBCL.

Upregulation of PD-1 has contributed to the suppressive role of LCLs on T cells function through the interaction with PD-L1 on LCLs, suggesting a negative regulation on the proliferation of T cells and secretion of cytokines. Based on our screen on the expression of PD-L1 in different tumor cell lines as well as primary samples, it is conceivable that different subtypes of lymphoma cells will have distinct intrinsic ability of modulating T cells mediated immune response. This is confirmed experimentally by our data that PD-1 pathway blockade could largely restore the T cell proliferation and secretion of cytokines in PD-L1 expressing EBV+DLBCL and ABC-DLBCL versus PD-L1-negative EBV-DLBCL and GCB-DLBCL. This raises a strategy that in the future patients can be stratified based on the expression of PD-L1, and then selectively treated with PD-1 blockade. Further studies will be required to confirm whether PD-1 blockade results in similar effects in primary DLBCL specimens from lymph nodes or other extranodal sites of tumor. Notably, PD-L1 in tumor microenvironment may play a role in host immune suppression through expression by non-tumor cells. It has been reported that 38% of PD-L1-negative DLBCLs were infiltrated by PD-L1+histiocytes [16]. Therefore, further study is needed to investigate the relationship between PD-L1-expressed histiocytes and T cells. Alternatively, PD-1-positive T cells might also express other inhibitory receptors such as CTLA-4, TIM-3, LAG3, and BTLA [21,42], suggesting that blocking of PD-1 alone might not fully restore the function of antitumor T cells [43,44], consistent with our results. Targeting multiple pathways may present a more efficient way in reversing T-cell exhaustion, and thus enhance the endogenous antitumor T-cell responses and improve outcome in EBV+DLBCL. The combination of PD-1/PD-L1 blockade antibody with other immune-stimulatory agents such as vaccines [45], Toll-like receptor ligands [46], or lenalidomide [47] has been proposed.

Taken together, combined with other preclinical studies showing that PD-1 blockade might also enhance tumor responses to other forms of immunotherapy [48,49], we reveal that PD-1/PD-L1 blockade presents a promising immunotherapeutic approach for EBV+DLBCL. These findings have clinical implications for designing personalized cancer immunotherapy to DLBCL.

## Supporting Information

S1 Fig PD-L1 expression on a panel of lymphoma cell lines. Flow cytometric analysis of PD-L1 expression in lymphoma cell lines is shown for some NHL cell lines. PD-L1 expression is a feature of EBV+ cell line, some ABC(non-GCB)-DLBCL (OCI-Ly-10 and HBL-1), ALCL (Kapras299) and T-ALL (Jurkat) but not of GCB-DLBCL (SU-DHL-4 and SU-DHL-6) and Burkitt lymphoma (Raji and Daudi).
(TIF)

S2 Fig PD-L1 expression in primary DLBCL tissue specimens. Histograms show PD-L1 expression on tumor B cells in freshly isolated cell suspensions from the tissue types indicated.
(TIF)

S3 Fig PD-1 blockade enhances the activation of T cells cocultured with allogeneic lymphoma cells (OCI-Ly-10, LCL and SU-DHL-4). Irradiated lymphoma cells, were stimulated for 1 week with T cells from healthy donors and then incubated with freshly irradiated target cells in the presence of media alone, anti-PD-1 antibody, or control antibody. After 4 days, supernatants were collected to analysis of IL-2,IFN-g,TNF-a and IL-10. Cytokines in supernatants were measured with cytometric beads array(CBA) by flowcytometry.
(TIF)

S1 Table Status of EBER expression and types of latency pattern on EBV positive cases. Note: 1:20% EBER+ tumor cells as a positive cut-off value. Abbreviations:ISH: in situ hybridization; EBER: EBV-encoded small nuclear RNA; IHC: immunohistochemistry; LMP: latent membrane protein; EBNA: Epstein-Barr nuclear antigen; ND: not determined.
(DOC)

S2 Table The ratio of CD4+ and CD8+ effector T cells and the ratio of PD-1 expression (%) on CD4+and CD8+ T cells in primary tissue and peripheral blood of GCB-DLBCL patients. Abbreviations:Tem: effector/memory T cell; LN: lymph node; PB: Peripheral blood.
(DOC)

S3 Table The ratio of CD4+ and CD8+ effector T cells and the ratio of PD-1 expression (%) on CD4+and CD8+ T cells in primary tissue and peripheral blood of ABC-DLBCL patients. Abbreviations:Tem: effector/memory T cell; LN: lymph node; PB: Peripheral blood.
(DOC)

S4 Table The ratio of CD4+ and CD8+ effector T cells and the ratio of PD-1 expression (%) on CD4+and CD8+ T cells in primary tissue and peripheral blood of EBV+DLBCL patients. Abbreviations:Tem: effector/memory T cell; LN: lymph node; PB: Peripheral blood.
(DOC)

## References

[1] de Visser KE, Eichten A, Coussens LM. Paradoxical roles of the immune system during cancer development. Nat Rev Cancer 2006;6:24–37. 16397525 10.1038/nrc1782

[2] Shankaran V, Ikeda H, Bruce AT, White JM, Swanson PE, Old LJ, et al IFNgamma and lymphocytes prevent primary tumour development and shape tumour immunogenicity. Nature 2001;410:1107–1111. 11323675 10.1038/35074122

[3] Bryan LJ, Gordon LI. Blocking tumor escape in hematologic malignancies: The anti-PD-1 strategy. Blood Rev 2015;29(1):25–32. 25260226 10.1016/j.blre.2014.09.004

[4] Ok CY1, Papathomas TG, Medeiros LJ, Young KH. EBV-positive diffuse large B-cell lymphoma of the elderly. Blood 2013;122: 328–340. 10.1182/blood-2013-03-489708 23649469 PMC3779382

[5] Francisco LM, Sage PT, Sharpe AH. The PD-1 pathway in tolerance and autoimmunity. Immunol Rev 2010;236:219–242. 10.1111/j.1600-065X.2010.00923.x 20636820 PMC2919275

[6] Kuchroo VK, Anderson AC, Petrovas C. Coinhibitory receptors and CD8 T cell exhaustion in chronic infections. Curr Opin HIV AIDS 2014;9:439–445. 25010894 10.1097/COH.0000000000000088

[7] Barber DL, Wherry EJ, Masopust D, Zhu B, Allison JP, Sharpe AH, et al Restoring function in exhausted CD8 T cells during chronic viral infection. Nature 2006;439:682–687. 16382236 10.1038/nature04444

[8] Jacobsen ED. Restoring antitumor immunity via PD-1 blockade after autologous stem-cell transplantation for diffuse large B-cell lymphoma. J Clin Oncol 2013;31:4268–4270. 10.1200/JCO.2013.51.7680 24127445

[9] Carreras J, Lopez-Guillermo A, Roncador G, Villamor N, Colomo L, Martinez A, et al High numbers of tumor-infiltrating programmed cell death 1-positive regulatory lymphocytes are associated with improved overall survival in follicular lymphoma. J Clin Oncol 2009;27:1470–1476. 10.1200/JCO.2008.18.0513 19224853

[10] Tzankov A, Meier C, Hirschmann P, Went P, Pileri SA, Dirnhofer S. Correlation of high numbers of intratumoral FOXP3+ regulatory T cells with improved survival in germinal center-like diffuse large B-cell lymphoma, follicular lymphoma and classical Hodgkin's lymphoma. Haematologica 2008;93:193–200. 10.3324/haematol.11702 18223287

[11] Muenst S, Hoeller S, Dirnhofer S, Tzankov A. Increased programmed death-1+ tumor-infiltrating lymphocytes in classical Hodgkin lymphoma substantiate reduced overall survival. Hum Pathol 2009;40:1715–1722. 10.1016/j.humpath.2009.03.025 19695683

[12] Chen L. Co-inhibitory molecules of the B7-CD28 family in the control of T-cell immunity. Nat Rev Immunol 2004;4:336–347. 15122199 10.1038/nri1349

[13] Armand P, Nagler A, Weller EA, Devine SM, Avigan DE, Chen YB, et al Disabling immune tolerance by programmed death-1 blockade with pidilizumab after autologous hematopoietic stem-cell transplantation for diffuse large B-cell lymphoma: results of an international phase II trial. J Clin Oncol 2013;31:4199–4206. 10.1200/JCO.2012.48.3685 24127452 PMC4878008

[14] Westin JR, Chu F, Zhang M, Fayad LE, Kwak LW, Fowler N, et al Safety and activity of PD1 blockade by pidilizumab in combination with rituximab in patients with relapsed follicular lymphoma: a single group, open-label, phase 2 trial. Lancet Oncol 2014;15:69–77. 10.1016/S1470-2045(13)70551-5 24332512 PMC3922714

[15] Dong H, Strome SE, Salomao DR, Tamura H, Hirano F, Flies DB, et al Tumor-associated B7-H1 promotes T-cell apoptosis:a potential mechanism of immune evasion. Nat Med 2002;8:793–800. 12091876 10.1038/nm730

[16] Andorsky DJ, Yamada RE, Said J, Pinkus GS, Betting DJ, Timmerman JM. Programmed Death Ligand 1 Is Expressed by Non-Hodgkin Lymphomas and Inhibits the Activity of Tumor-Associated T Cells. Clin Cancer Res 2011;17:4232–4244. 10.1158/1078-0432.CCR-10-2660 21540239

[17] Chen BJ, Chapuy B, Ouyang J, Sun HH, Roemer MG, Xu ML, et al PD-L1 Expression Is Characteristic of a Subset of Aggressive B-cell Lymphomas and Virus-Associated Malignancies. Clin Cancer Res 2013;19:3462–3473. 10.1158/1078-0432.CCR-13-0855 23674495 PMC4102335

[18] Hui-Yuen J, McAllister S, Koganti S, Hill E, Bhaduri-McIntosh S. Establishment of Epstein-Barr virus growth-transformed lymphoblastoid cell lines. J Vis Exp 2011;8. 10.3791/3321 PMC3308597 22090023

[19] IARC. WHO classification of tumours of haematopoietic and lymphoid tissues, 4th edn.,vol. 2 Lyon: WHO Press, 2008.

[20] Hans CP, Weisenburger DD, Greiner TC, Gascoyne RD, Delabie J, Ott G, et al Confirmation of the molecular classification of diffuse large B-cell lymphoma by immunohistochemistry using a tissue microarray. Blood 2004;103:275–282. 14504078 10.1182/blood-2003-05-1545

[21] Lu CH, Lee KF, Chen CC, Chen YY, Huang CE, Tsai PS, et al Clinical characteristics and treatment outcome in a Taiwanese population of patients with Epstein-Barr virus-positive diffuse large B-cell lympho-ma. Jpn J Clin Oncol 2014; 44:1164–1171. 10.1093/jjco/hyu155 25320341

[22] Oyama T, Yamamoto K, Asano N, Oshiro A, Suzuki R, Kagami Y, et al Age-related EBV-associated B-Cell lymphoproliferative disorders constitute a distinct clinicopathologic group: A study of 96 patients. Clin Cancer Res 2007;13:5124–5132. 17785567 10.1158/1078-0432.CCR-06-2823

[23] Quah BJ, Parish CR. The use of carboxyfluorescein diacetate succinimidyl ester (CFSE) to monitor lymphocyte proliferation. J Vis Exp 2010;12–44. 10.3791/2259 PMC3185625 20972413

[24] Pardoll DM. The blockade of immune checkpoints in cancer immunotherapy. Nat Rev Cancer 2012;12:252–264. 10.1038/nrc3239 22437870 PMC4856023

[25] Blackburn SD, Shin H, Haining WN, Zou T, Workman CJ, Polley A, et al Coregulation of CD8+ T cell exhaustion by multiple inhibitory receptors during chronic viral infection. Nat Immunol 2009;10:29–37. 10.1038/ni.1679 19043418 PMC2605166

[26] Baitsch L, Baumgaertner P, Devêvre E, Raghav SK, Legat A, Barba L, et al Exhaustion of tumor-specific CD8(+) T cells in metastases from melanoma patients. J Clin Invest 2011;121:2350–2360. 10.1172/JCI46102 21555851 PMC3104769

[27] Yang ZZ, Novak AJ, Stenson MJ, Witzig TE, Ansell SM. Intratumoral CD4+CD25+ Regulatory T-cell-mediated suppression of infiltrating CD4+ T cells in B-cell non-Hodgkin lymphoma. Blood 2006;107:3639–3946. 16403912 10.1182/blood-2005-08-3376 PMC1895773

[28] Chen DS, Irving BA, Hodi FS. Molecular pathways: next-generation immunotherapy-inhibiting programmed death-ligand 1 and programmed death-1. Clin Cancer Res 2012;18(24):6580–6587 10.1158/1078-0432.CCR-12-1362 23087408

[29] Gao Q, Wang XY, Qiu SJ, Yamato I, Sho M, Nakajima Y, et al Overexpression of PD-L1 significantly associates with tumor aggressiveness and postoperative recurrence in human hepatocellular carcinoma. Clin Cancer Res 2009;15(3):971–979 10.1158/1078-0432.CCR-08-1608 19188168

[30] Hino R, Kabashima K, Kato Y, Yagi H, Nakamura M, Honjo T, et al Tumor cell expression of programmed cell death-1 ligand 1 is a prognostic factor for malignant melanoma. Cancer 2010;116(7):1757–1766 10.1002/cncr.24899 20143437

[31] Mu CY, Huang JA, Chen Y, Chen C, Zhang XG. High expression of PD-L1 in lung cancer may contribute to poor prognosis and tumor cells immune escape through suppressing tumor infiltrating dendritic cells maturation. Med Oncol 2011;28(3):682–688 10.1007/s12032-010-9515-2 20373055

[32] Green MR, Rodig S, Juszczynski P, Ouyang J, Sinha P, O’Donnell E, et al Constitutive AP-1 activity and EBV infection induce PD-L1 in Hodgkin lymphomas and posttransplant lymphoproliferative disorders: implications for targeted therapy. Clin Cancer Res 2012; 18(6):1611–1618. 10.1158/1078-0432.CCR-11-1942 22271878 PMC3321508

[33] Wang BJ, Bao JJ, Wang JZ, Wang Y, Jiang M, Xing MY, et al Immunostaining of PD-1/PD-Ls in liver tissues of patients with hepatitis and hepatocellular carcinoma. World J Gastroenterol 2011; 17(28):3322–3329. 10.3748/wjg.v17.i28.3322 21876620 PMC3160536

[34] Lyford-Pike S, Peng S, Young GD, Taube JM, Westra WH, Akpeng B, et al Evidence for a role of the PD-1:PD-L1 pathway in immune resistance of HPV-associated head and neck squamous cell carcinoma. Cancer Res 2013; 73(6):1733–1741. 10.1158/0008-5472.CAN-12-2384 23288508 PMC3602406

[35] Badoual C, Hans S, Merillon N, Van Ryswick C, Ravel P, Benhamouda N, et al PD-1-expressing tumor-infiltrating T cells are a favorable prognostic biomarker in HPV-associated head and neck cancer. Cancer Res 2013; 73(1):128–138. 10.1158/0008-5472.CAN-12-2606 23135914

[36] Fang W, Zhang J, Hong S, Zhan J, Chen N, Qin T, et al EBV-driven LMP1 and IFN-γ up-regulate PD-L1 in nasopharyngeal carcinoma: Implications for oncotargeted therapy. Oncotarget 2014;5(23):12189–12202. 25361008 10.18632/oncotarget.2608 PMC4322961

[37] Green MR, Monti S, Rodig SJ, Juszczynski P, Currie T, O'Donnell E, et al Integrative analysis reveals selective 9p24.1 amplification, increased PD-1 ligand expression, and further induction via JAK2 in nodular sclerosing Hodgkin lymphoma and primary mediastinal large B-cell lymphoma. Blood 2010;116:3268–3277. 10.1182/blood-2010-05-282780 20628145 PMC2995356

[38] Marzec M, Zhang Q, Goradia A, Raghunath PN, Liu X, Paessler M, et al Oncogenic kinase NPM/ALK induces through STAT3 expression of immunosuppressive protein CD274 (PD-L1, B7-H1). Proc Natl Acad Sci U S A 2008; 105(52):20852–20857. 10.1073/pnas.0810958105 19088198 PMC2634900

[39] Dong H, Zhu G, Tamada K, Chen L. B7-H1, a third member of the B7 family, co-stimulates T-cell proliferation and interleukin-10 secretion. Nat Med 1999;5:1365–1369. 10581077 10.1038/70932

[40] Shinohara T, Taniwaki M, Ishida Y, Kawaichi M, Honjo T. Structure and chromosomal localization of the human PD-1 gene (PDCD1). Genomics 1994;23:704–706. 7851902 10.1006/geno.1994.1562

[41] Hamilton-Dutoit SJ, Rea D, Raphael M, Sandvej K, Delecluse HJ, Gisselbrecht C, et al Epstein–Barr virus-latent gene expression and tumor cell phenotype in acquired immunodeficiency syndrome-related non-Hodgkin's lymphoma. Correlation of lymphoma phenotype with three distinct patterns of viral latency. Am J Pathol 1993;143:1072–1085. 8214003 PMC1887058

[42] Wherry EJ.T cell exhaustion.Nat Immunol 2011;12:492–499. 21739672 10.1038/ni.2035

[43] Ansell SM, Hurvitz SA, Koenig PA, LaPlant BR, Kabat BF, Fernando D, et al Phase I study of ipilimumab, an anti-CTLA-4 monoclonal antibody, in patients with relapsed and refractory B-cell non-Hodgkin lymphoma. Clin Cancer Res 2009;15:6446–6453. 10.1158/1078-0432.CCR-09-1339 19808874 PMC2763019

[44] Wolchok JD, Kluger H, Callahan MK, Postow MA, Rizvi NA, Lesokhin AM, et al Nivolumab plus ipilimumab in advanced melanoma. N Engl J Med 2013;369:122–133. 10.1056/NEJMoa1302369 23724867 PMC5698004

[45] Schuster SJ, Neelapu SS, Gause BL, Janik JE, Muggia FM, Gockerman JP, et al Vaccination with patient specific tumor-derived antigen in first remission improves disease-free survival in follicular lymphoma. J Clin Oncol 2011;29:2787–2794. 10.1200/JCO.2010.33.3005 21632504 PMC3139394

[46] Houot R, Levy R. T-cell modulation combined with intratumoral CpG cures lymphoma in a mouse model without the need for chemotherapy. Blood 2009; 113: 3546–3552. 10.1182/blood-2008-07-170274 18941113 PMC2668854

[47] Houot R, Goldstein MJ, Kohrt HE, Myklebust JH, Alizadeh AA, Lin JT, et al Therapeutic effect of CD137 immunomodulation in lymphoma and its enhancement by Treg depletion. Blood 2009; 114:3431–3438. 10.1182/blood-2009-05-223958 19641184 PMC2765679

[48] Curran MA, Montalvo W, Yagita H, Allison JP. PD-1 and CTLA-4 combination blockade expands infiltrating T cells and reduces regulatory T and myeloid cells within B16 melanoma tumors. Proc Natl Acad Sci U S A 2010;107(9):4275–4280. 10.1073/pnas.0915174107 20160101 PMC2840093

[49] Zhou Q, Xiao H, Liu Y, Peng Y, Hong Y, Yagita H, et al Blockade of programmed death-1 pathway rescues the effector function of tumor-infiltrating T cells and enhances the antitumor efficacy of lentivector immunization. J Immunol 2010;185(9):5082–5092. 10.4049/jimmunol.1001821 20926790 PMC3077721

