# Supplementary figures and images for: PD-1 Blockade Can Restore Functions of T-Cells in Epstein-Barr Virus-Positive Diffuse Large B-Cell Lymphoma In Vitro
Source: PLoS One. 2015 Sep 11;10(9):e0136476. doi: 10.1371/journal.pone.0136476 (PMC4567291; doi:10.1371/journal.pone.0136476)

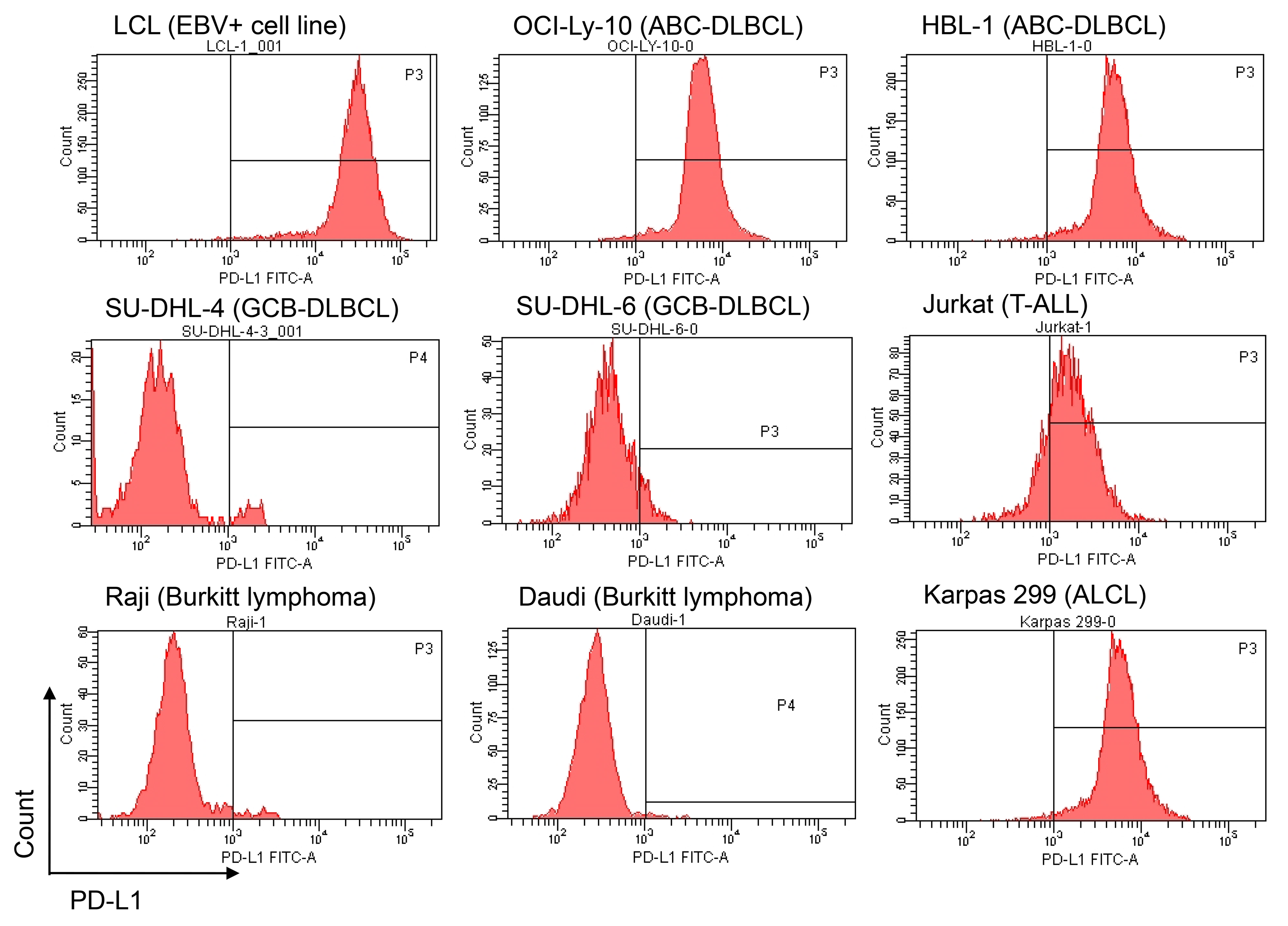

Supplement: S1 Fig — Flow cytometric analysis of PD-L1 expression in lymphoma cell lines is shown for some NHL cell lines. PD-L1 expression is a feature of EBV+ cell line, some ABC(non-GCB)-DLBCL (OCI-Ly-10 and HBL-1), ALCL (Kapras299) and T-ALL (Jurkat) but not of GCB-DLBCL (SU-DHL-4 and SU-DHL-6) and Burkitt lymphoma (Raji and Daudi). (TIF) [file pone.0136476.s001.tif]

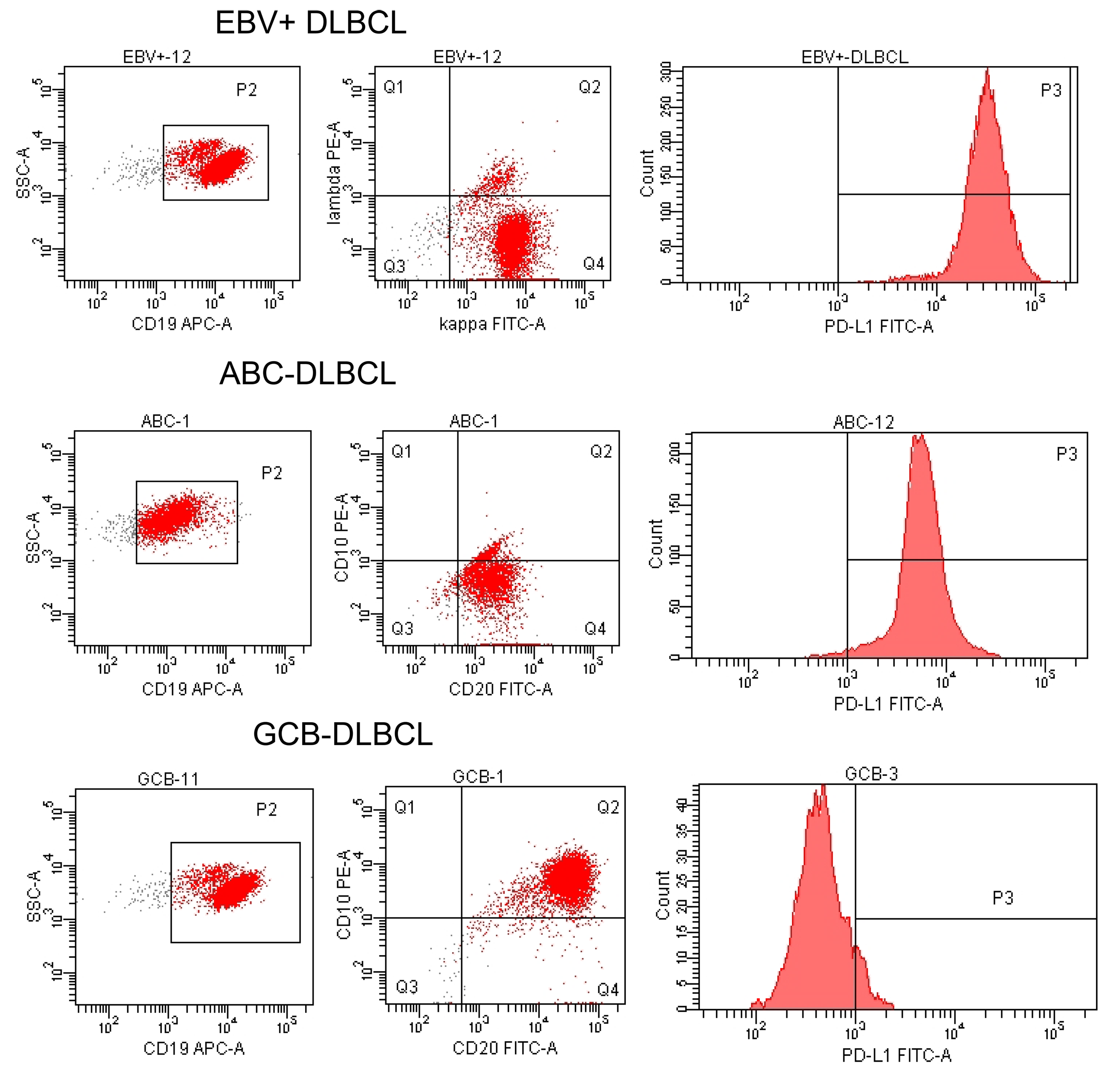

Supplement: S2 Fig — Histograms show PD-L1 expression on tumor B cells in freshly isolated cell suspensions from the tissue types indicated. (TIF) [file pone.0136476.s002.tif]

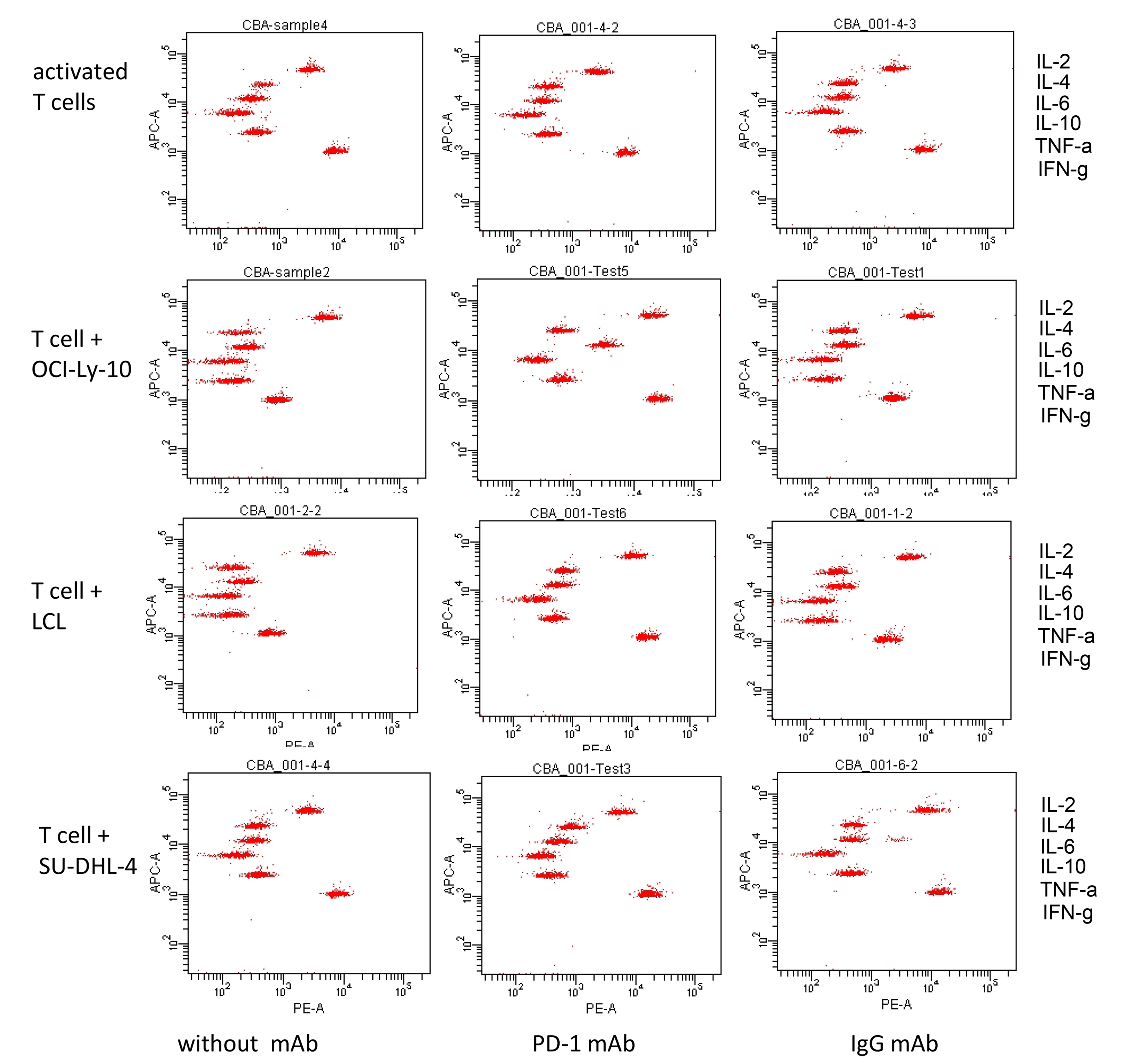

Supplement: S3 Fig — Irradiated lymphoma cells, were stimulated for 1 week with T cells from healthy donors and then incubated with freshly irradiated target cells in the presence of media alone, anti-PD-1 antibody, or control antibody. After 4 days, supernatants were collected to analysis of IL-2,IFN-g,TNF-a and IL-10. Cytokines in supernatants were measured with cytometric beads array(CBA) by flowcytometry. (TIF) [file pone.0136476.s003.tif]
