# Supplementary material for: PD-1 Blockade Can Restore Functions of T-Cells in Epstein-Barr Virus-Positive Diffuse Large B-Cell Lymphoma In Vitro
Source: PLoS One. 2015 Sep 11;10(9):e0136476. doi: 10.1371/journal.pone.0136476 (PMC4567291; doi:10.1371/journal.pone.0136476)
Supplement: S1 Table — Note: 1:20% EBER+ tumor cells as a positive cut-off value. Abbreviations:ISH: in situ hybridization; EBER: EBV-encoded small nuclear RNA; IHC: immunohistochemistry; LMP: latent membrane protein; EBNA: Epstein-Barr nuclear antigen; ND: not determined. (DOC) [file pone.0136476.s004.doc]

**S1 Table.Status of EBER expression and types of latency pattern on EBV positive cases**

| Patient No. | ISH | | | IHC | | | |  |
| --- | --- | --- | --- | --- | --- | --- | --- | --- |
|  | EBER | Positive cell (%)1 | LMP1 | | LMP2A | EBNA2 | EBNA3A | Latency  pattern |
| ED-pt1 | + | 40 | + | | - | + | + | III |
| ED-pt2 | + | 70 | + | | - | + | + | III |
| ED-pt3 | + | 30 | + | | - | + | + | III |
| ED-pt4 | + | 60 | + | | ND | ND | ND | II/III |
| ED-pt5 | + | 80 | + | | ND | ND | ND | II/III |
| ED-pt6 | + | 70 | + | | - | + | + | III |
| ED-pt7 | + | 50 | - | | - | + | + | III |

Note: 1:20% EBER+ tumor cells as a positive cut-off value.

Abbreviations:ISH: in situ hybridization; EBER: EBV-encoded small nuclear RNA; IHC: immunohistochemistry; LMP: latent membrane protein; EBNA: Epstein-Barr nuclear antigen; ND: not determined.
