# Supplementary material for: PD-1 Blockade Can Restore Functions of T-Cells in Epstein-Barr Virus-Positive Diffuse Large B-Cell Lymphoma In Vitro
Source: PLoS One. 2015 Sep 11;10(9):e0136476. doi: 10.1371/journal.pone.0136476 (PMC4567291; doi:10.1371/journal.pone.0136476)
Supplement: S2 Table — Abbreviations:Tem: effector/memory T cell; LN: lymph node; PB: Peripheral blood. (DOC) [file pone.0136476.s005.doc]

**S2 Table . The ratio of CD4+ and CD8+ effector T cells and the ratio of PD-1 expression (%) on CD4+and CD8+ T cells in primary tissue and peripheral blood of GCB-DLBCL patients**

| GCB-  DLBCL(n=8) | CD4 Tem/CD4+T  (%) | | CD8 Tem/CD8+T  (%) | | PD-1/CD4+T cells  (%) | | PD-1/CD8+T cells  (%) | |
| --- | --- | --- | --- | --- | --- | --- | --- | --- |
|  | LN | PB | LN | PB | LN | PB | LN | PB |
| GD-pt1 | 53.8 | 26.5 | 57.7 | 25.1 | 52.9 | 47.2 | 66.1 | 41.2 |
| GD-pt2 | 67.1 | 37.1 | 54.6 | 38.1 | 49.6 | 25.7 | 59.1 | 39.9 |
| GD-pt3 | 61.8 | 32.5 | 71.3 | 46.8 | 59.6 | 33.6 | 77.8 | 36.7 |
| GD-pt4 | 52.3 | 29.0 | 56.8 | 27.2 | 63.7 | 42.7 | 69.9 | 44.2 |
| GD-pt5 | 60.6 | 35.0 | 71.2 | 43.9 | 41.9 | 35.5 | 54.3 | 30.2 |
| GD-pt6 | 59.3 | 25.1 | 64.8 | 48.5 | 67.6 | 48.9 | 74.3 | 50.7 |
| GD-pt7 | 64.7 | 33.3 | 68.0 | 29.5 | 45.9 | 38.5 | 46.3 | 33.4 |
| GD-pt8 | 70.0 | 39.9 | 77.2 | 32.9 | 57.1 | 28.7 | 50.2 | 47.5 |
| mean | 61.20±6.13 | 32.30±5.15 | 65.20±8.14 | 36.50±9.15 | 54.79±8.85 | 37.60±8.35 | 62.25±11.55 | 40.48±6.95 |

Abbreviations:Tem: effector/memory T cell; LN: lymph node; PB: Peripheral blood.
