# Supplementary material for: PD-1 Blockade Can Restore Functions of T-Cells in Epstein-Barr Virus-Positive Diffuse Large B-Cell Lymphoma In Vitro
Source: PLoS One. 2015 Sep 11;10(9):e0136476. doi: 10.1371/journal.pone.0136476 (PMC4567291; doi:10.1371/journal.pone.0136476)
Supplement: S3 Table — Abbreviations:Tem: effector/memory T cell; LN: lymph node; PB: Peripheral blood. (DOC) [file pone.0136476.s006.doc]

**S3 Table. The ratio of CD4+ and CD8+ effector T cells and the ratio of PD-1 expression (%) on CD4+and CD8+ T cells in primary tissue and peripheral blood of ABC-DLBCL patients**

| ABC-  DLBCL  (n=12) | CD4 Tem/CD4+T  (%) | | CD8 Tem/CD8+T  (%) | | PD-1/CD4+T cells  (%) | | PD-1/CD8+T cells  (%) | |
| --- | --- | --- | --- | --- | --- | --- | --- | --- |
|  | LN | PB | LN | PB | LN | PB | LN | PB |
| AD-pt1 | 74.4 | 31.2 | 86.2 | 44.7 | 67.0 | 31.8 | 51.9 | 55.9 |
| AD-pt2 | 45.8 | 47.7 | 61.2 | 50.0 | 54.8 | 51.9 | 78.2 | 44.6 |
| AD-pt3 | 53.4 | 25.2 | 83.2 | 34.7 | 76.9 | 34.2 | 80.8 | 54.4 |
| AD-pt4 | 50.6 | 40.2 | 75.5 | 46.4 | 43.2 | 53.2 | 59.4 | 43.9 |
| AD-pt5 | 79.3 | 27.3 | 69.3 | 36.5 | 72.1 | 35.5 | 54.1 | 47.7 |
| AD-pt6 | 54.2 | 46.3 | 74.7 | 31.7 | 47.5 | 56.7 | 62.4 | 60.1 |
| AD-pt7 | 60.7 | 29.4 | 50.2 | 29.4 | 40.9 | 37.8 | 85.3 | 43.3 |
| AD-pt8 | 48.4 | 24.8 | 66.4 | 47.8 | 70.4 | 47.0 | 65.0 | 56.0 |
| AD-pt9 | 67.3 | 42.1 | 63.1 | 32.9 | 45.7 | 41.1 | 72.5 | 52.2 |
| AD-pt10 | 82.1 | 42.9 | 80.3 | 38.1 | 50.8 | 49.6 | 56.8 | 43.2 |
| AD-pt11 | 77.1 | 33.5 | 55.1 | 41.3 | 74.6 | 46.3 | 75.3 | 55.7 |
| AD-pt12 | 73.6 | 37.9 | 53.4 | 42.8 | 63.0 | 39.4 | 84.1 | 39.3 |
| mean | 63.91±13.20 | 35.71±8.19 | 68.22±12.00 | 39.69±6.79 | 58.91±13.20 | 43.71±8.19 | 68.82±12.00 | 49.69±6.78 |

Abbreviations:Tem: effector/memory T cell; LN: lymph node; PB: Peripheral blood.
