# Supplementary material for: PD-1 Blockade Can Restore Functions of T-Cells in Epstein-Barr Virus-Positive Diffuse Large B-Cell Lymphoma In Vitro
Source: PLoS One. 2015 Sep 11;10(9):e0136476. doi: 10.1371/journal.pone.0136476 (PMC4567291; doi:10.1371/journal.pone.0136476)
Supplement: S4 Table — Abbreviations:Tem: effector/memory T cell; LN: lymph node; PB: Peripheral blood. (DOC) [file pone.0136476.s007.doc]

**S4 Table. The ratio of CD4+ and CD8+ effector T cells and the ratio of PD-1 expression (%) on CD4+and CD8+ T cells in primary tissue and peripheral blood of EBV+DLBCL patients**

| EBV+  DLBCL  (n=7) | CD4 Tem/CD4+T  (%) | | CD8 Tem/CD8+T  (%) | | PD-1/CD4+T cells  (%) | | PD-1/CD8+T cells  (%) | |
| --- | --- | --- | --- | --- | --- | --- | --- | --- |
|  | LN | PB | LN | PB | LN | PB | LN | PB |
| ED-pt1 | 72.5 | 42.7 | 82.4 | 53.7 | 58.8 | 46.8 | 72.5 | 42.4 |
| ED-pt2 | 52.7 | 50.3 | 66.5 | 36.9 | 71.4 | 43.5 | 68.4 | 53.9 |
| ED-pt3 | 63.1 | 32.9 | 83.5 | 39.3 | 55.3 | 50.1 | 85.8 | 49.8 |
| ED-pt4 | 55.6 | 40.1 | 93.7 | 33.7 | 70.3 | 38.4 | 82.8 | 56.4 |
| ED-pt5 | 90.7 | 35.7 | 92.1 | 49.2 | 67.1 | 31.9 | 59.9 | 38.9 |
| ED-pt6 | 87.3 | 46.2 | 64.1 | 52.2 | 48.9 | 35.8 | 63.4 | 45.9 |
| ED-pt7 | 80.2 | 53.1 | 71.9 | 45.1 | 80.4 | 29.3 | 89.6 | 35.3 |
| mean | 71.73±15.12 | 43.00±7.41 | 79.17±11.89 | 44.30±7.83 | 64.60±10.81 | 39.40±7.73 | 74.63±11.56 | 46.08±7.78 |

Abbreviations:Tem: effector/memory T cell; LN: lymph node; PB: Peripheral blood.
